# Resina Draconis Particles Encapsulated in a Hyaluronic-Acid-Based Hydrogel to Treat Complex Burn Wounds

**DOI:** 10.3390/pharmaceutics14102087

**Published:** 2022-09-29

**Authors:** Lijun Xu, Ziqiang Zhou, Yuying Chen, Huangjie Lu, Ping Hu

**Affiliations:** College of Pharmacy, Jinan University, Guangzhou 511436, China

**Keywords:** hydrogel, Resina Draconis, burn, wound healing, hyaluronic acid, Schiff base, topical drug delivery

## Abstract

Severe burns require urgent new dressing treatments due to their irregular wounds and secondary injuries associated with dressing changes. In this study, a hyaluronic-acid-based hydrogel was developed to treat complex burn wounds. This hydrogel was prepared by mixing and cross-linking oxidized hyaluronic acid (OHA) and carboxymethyl chitosan (CMCS) through Schiff base reactions. Micronized Resina Draconis particles were encapsulated in this hydrogel to achieve sustained release of the active components when applied on wounds. The Resina-Draconis-loaded hydrogel (RD-Gel) demonstrated good mechanical properties and excellent self-healing. The results of in vitro experiments confirmed that RD-Gel had good biocompatibility, and was able to enhance cell migration and inhibit the production of inflammatory cytokines. It also induced rapid hemostasis in rats, downregulated the levels of inflammatory cytokines, and promoted collagen regeneration on model animals, eventually accelerating the rebuilding of skin structures and wound recovery.

## 1. Introduction

Burns are tissue damage caused by heat, including from hot liquids (water, soup, oil, etc.), vapors, hot gases, flames, and incendiary metallic liquids or solids (such as steel) [1]. Severe burns can cause a variety of complications and place a significant burden on families and medical care [2,3]. Over the past 50 years, the treatment of burns has constantly been updated, with the application of dressings representing a major adjunct to promote wound healing [4,5,6]. Traditional dressings such as gauze, transparent film, and foam have a limited scope of action due to their limited ability to absorb exudate and the high risk of secondary injury from dressing changes [7,8], clinically no longer satisfied with a single functional dressing either. As the chronic wound treatment system has evolved, it has increasingly been recognized that balancing skin moisture levels is important and that a suitably moist environment appears to be more conducive to wound healing than an absolutely dry wound bed [9]. Furthermore, an ideal dressing would be able to provide substantial therapeutic effect to promote the recovery of wounds [10]. Therefore, it is especially essential to find a multifunctional dressing to treat burns and accelerate healing.

Resina Draconis, the resin of the sword-leaved dragon’s blood tree of the Liliaceae family, is a traditional and valuable herb widely distributed in Yunnan and Guangxi provinces in China and other southeastern Asian countries [11]. It has been used for hundreds of years for the treatment of bruises and pain. Recently, it has been discovered that Resina Draconis contains several pharmacologically active compounds, such as Loureirin A, Loureirin B and astragalus, etc., which exhibit antioxidant, anti-inflammatory, hemostatic, and anti-ulcer effects [12,13,14]. However, the topical applications of Resina Draconis were usually used in the form of coarse powders, which lacked sufficient exposure of the active components on the wound due to the inadequate release of such components from the relatively large particles of the Resina Draconis. Correspondingly, the efficacy and the mechanisms of Resina Draconis as a topical pharmaceutical agent are also less well explored.

Hydrogels are three-dimensional networks of hydrophilic polymer chains held together by cross-linking [15]. As biomaterials closest to a living tissue, they generally have good biocompatibility and can be in direct contact with human tissue. The porous microstructure allows the hydrogel to absorb excess exudate and keep the wound moist, transport oxygen and nutrients, and promote wound healing. Hydrogels can also act as carriers to transport drugs to the wound bed [16]. Moreover, building polymeric hydrogel networks based on advanced cross-linking structures would be very beneficial. Schiff base, as a typical click chemistry reaction, can be dynamic and reversible under mild conditions, Schiff base cross-linking between macromolecules can confer self-healing properties to the material, which represents one of the most valuable methods for preparing smart biocompatible hydrogels [17,18,19].

**Scheme 1:**
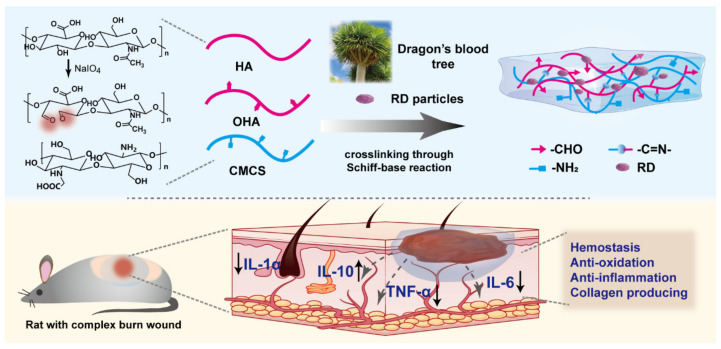
Schematic representations of RD-Gel hydrogel for burn wounds. The increase in anti-inflammatory cytokine IL-10 and the decreases in inflammatory cytokines including IL-1α, TNF-α, and IL-6 were expected after applying the RD-Gel on rats. OHA: oxidized hyaluronic acid, CMCS: carboxymethyl chitosan, RD: Resina Draconis.

In this study, we propose the preparation a biocompatible hydrogel dressing using oxidized hyaluronic acid and carboxymethyl chitosan cross-linked through Schiff base reaction. As shown in Scheme 1, micronized Resina Draconis particles were loaded in the hydrogel to guarantee uniform distribution of the particles and the sustained release of the effective components when applied on the wounds. OHA is an oxidized derivative of HA, which consists of repeated disaccharide units of β-D-glucuronic acid and N-acetyl-D-glucosamine; the abundant carboxyl and hydroxyl groups confer a high hydrophilicity to HA. It is widely found in human skin and connective tissue and has good biocompatibility with the skin [20]. It also serves as a component of the extracellular matrix and binds to CD44 receptors to regulate re-epithelialization [21]. Carboxymethyl chitosan (CMCS) is also widely used in wound dressings due to its good water solubility and biodegradability [22]. Additionally, the amino groups on CMCS molecules react with the aldehyde groups on OHA chains to form imine bonds, which are critical cross-linking structures of the prepared hydrogel.

## 2. Materials and Methods

### 2.1. Materials

Hyaluronic acid (HA) (Mw = 10–20 kDa) was purchased from Macklin (Shanghai, China). Carboxymethyl chitosan (CMCS) was purchased from Macklin (Shanghai, China). Resina Draconis (RD) was purchased from Yun Nan China. Dulbecco’s modified Eagle’s medium (DMEM), RPMI-1640 medium, fetal bovine serum (FBS), Trypsin EDTA, and Penicillin–Streptomycin solution were purchased from Gibco (USA). Loureirin A and Loureirin B standard was purchased from Chengdu Herbpurify CO.LTD. Acetonitrile was of high-performance liquid chromatography (HPLC) grade. All other reagents and solvents were of analytical reagent grade.

### 2.2. Physicochemical Properties of Resina Draconis

#### 2.2.1. HPLC Conditions

Chromatography was performed on an Agilent 1290 Infinity LC system. Chromatographic separations were carried out on a SuperLuC18-AQ5u (4.6 mm × 250 mm, 5 μm, Guangzhou FLM Scientific Instrument Co. Ltd., Guangzhou, China). The column oven was maintained at 30 °C. The mobile phase was composed of acetonitrile (A) and 0.1% aqueous formic acid (B), and the flow rate was set at 1.0 mL/min following the gradient program: 46% (B), 54% acetonitrile (A) for 20 mins. The detection wavelength was 275 nm and the injection volume was 4 μL.

#### 2.2.2. Preparation of Standard Solution

The stock solutions of Loureirin A and Loureirin B reference substance were prepared to concentrations of 1.5 μg/mL, 7.5 μg/mL, 15 μg/mL, 30 μg/mL, and 60 μg/mL. The standard solutions were stored with protection from light. Using the chromatographic conditions in detailed in Section 2.2.1, the standard samples were measured and the standard curve was obtained using the integrated peak area value as the vertical coordinate (Y) and the standard concentration (μg/mL) as the horizontal coordinate (X).

#### 2.2.3. Micronizing Resina Draconis and Characterizations of the Particles

The Resina Draconis was crushed with a micronizer (Royalstar, RS-FS1406,) with different rounds to obtain particles with different sizes. Different batches of the particles were tested with a Malvern laser particle sizer to test the obtained particle size range. Scanning electronic microscopy (SEM, Hitachi SU1000, Ibarakiken, Japan) was used to visualize the particles. To measure the content of the active components in the particles, a portion of the particles (4.0 g) was dissolved in 4 mL of methanol. After sonicating, the particles were completely dissolved, and the resultant solution was filtered through a 0.22 μm nylon filter membrane. The filtrate was determined according to the chromatographic conditions detailed in Section 2.2.1 and the peak area was recorded.

### 2.3. Synthesis of OHA

To synthesize OHA, 1.0 g (2.5 mmol) HA was dissolved in 100 mL of double-distilled water (dd) at a concentration of 10 mg/mL. When HA was completely dissolved, 0.53 g (2.5 mmol) NaIO_4_ was added. The molar ratio of NaIO_4_ to HA was 1:1. The mixture was then stirred at normal temperature in the dark for 4 h. The solution was purified by exhaustive dialysis (MWCO 14000) using distilled water for 3 days after the reaction, and the water was changed at least once a day during the dialysis. The product was obtained by freeze drying. The actual degree of oxidation of HA was quantified by measuring the number of aldehyde groups in the polymer using a hydroxylamine hydro-chloride method. The chemical structures of lyophilized HA and OHA were verified using a Fourier-Transform infrared spectrometer (JASCO FT/IR-4600, Tokyo, Japan); ^1^H NMR spectra of HA and OHA were obtained on an Avance III 400 MHz NMR instrument using D_2_O as the solvent.

### 2.4. Preparations of Blank Hydrogels and Resina-Draconis-Loaded Hydrogels

Synthesized OHA was dissolved in phosphate-buffered saline (PBS) (10 mM, pH 7.4) to a final concentration of 4% (w/v). CMCS was dissolved in PBS to a final concentration of 4.5% (w/v). The volume ratio of OHA and CMCS solution was set at 1:3. The hydrogel was formed by vortexing and mixing. Formed hydrogel was confirmed by inverting the container for 30 s and shaking gently without collapsing. The Resina Draconis particles were loaded into the hydrogel by physical mixing. Subsequently, 5 g or 10 g of the Resina Draconis particles were homogeneously dispersed in the PBS solution of OHA, and vortexed with CMCS at a ratio of 1:3 to form a Resina-Draconis-loaded gel. The obtained blank hydrogel was named Gel. The obtained Resina Draconis hydrogels were named 0.5% wt. RD-Gel and 1% wt. RD-Gel.

### 2.5. Characterizations of Hydrogels

#### 2.5.1. Gelation and Physical Appearance of the Hydrogel

The gelation time of the hydrogel was determined using a vial inversion method. The precursor solutions were added to the vial and gelation was performed at 37 °C. The mobility of the mixture was observed, and the gelation time was recorded if the sample stopped flowing when the vial was inverted.

The Gel and RD-Gel hydrogels were examined through scanning electron microscopy. In brief, hydrogel specimens were lyophilized, cut into thin pieces, and sprayed with gold. The crossed section morphologies were visualized using SEM operated at an accelerating voltage of 15.0 kV.

#### 2.5.2. Injectable, Self-Healing Properties of the Hydrogel

Two circular shapes of hydrogels were stained with rhodamine B and methylene blue, followed by cutting the hydrogels from the middle with a knife and joining the differently colored semicircular hydrogels together to observe their self-healing. To investigate the injectable properties of the hydrogels, OHA and CMCS were premixed and loaded into a 5 mL syringe. The mixture was pushed out through the needle and the formed gel was observed and recorded.

#### 2.5.3. Swelling Property of the Hydrogel

The swelling behavior of hydrogels was evaluated. The weighed dry hydrogels (W_0_) were incubated in 20 mL of PBS (10 mM, pH 7.4) at 37 °C. At specified time intervals, the hydrogels (W_t_) were weighed after the removal of residual moisture on the gel surface with filter papers. Every test was repeated three times. The swelling rate was calculated according to the following formula:

The swelling rate (%) = (W_t_ − W_0_) /W_0_ × 100% (1)

where W_0_ is the mass of the dried hydrogel, and W_t_ is the mass of the hydrogel after swelling at different time.

#### 2.5.4. Rheological Characterizations

Rheological tests were performed on a Kinexus Lab+ Rheometer equipped with a 10 mm diameter parallel plate with a gap of 1 mm. A frequency sweep from 0.1 to 100 rad/s at a constant strain of 1% was conducted on the sample. The elastic modulus (G′) and viscous modulus (G″) were measured as a function of frequency at 25 °C. The critical strain of the hydrogel was measured using an oscillatory mode–amplitude sweep test. The hydrogels were exposed to strain amplitude effects ranging from 1% to 500%, with temperature and frequency maintained constant at 25 °C and 1 Hz. Different shear rates from 0.1 to 1000 s^−1^ were applied on the hydrogels. The viscosity at steady state was recorded. To investigate the self-healing properties of hydrogels, quantitative analysis of the effect of strain amplitude was performed. Hydrogels were subjected to strain amplitude effects from 1% (at 100 s intervals) to 300% for three cycles.

### 2.6. In Vitro Drug Release

Experiments of in vitro drug release of the hydrogels were performed in a constant-temperature oscillating incubator at 37 °C. RD-Gel samples were immersed in 3 mL of PBS containing 0.5% (v/v) Tween 80 (Ph = 7.4) and incubated in a shaking incubator at 37 °C and 100 rpm. At set time points, 1 mL of the medium was extracted, and the same volume of fresh medium was replenished. The contents of loureirin A and loureirin B in the samples were measured by the HPLC method mentioned above. The release profiles were plotted after the calculations using the standard curve described in Section 2.2.2 to determine the release behavior of RD-Gel.

### 2.7. The Hemocompatibility of the Hydrogel

Fresh rabbit blood was centrifuged at 4 °C, 3500 rpm, for 10 min, and the supernatant was discarded. The erythrocyte precipitate was repeatedly washed 3–4 times with sterile PBS buffer to be colorless and clear and diluted to a 5% (v/v) suspension. The RD-Gel extracts were prepared in PBS in a constant-temperature oscillating incubator at 37 °C for 48 h and diluted to different concentrations; subsequently, the erythrocyte suspension was inoculated in a 96-well plate at a density of 50 μL/well, and 50 μL of each concentration of the drug-loaded hydrogel extract was added to the well and incubated at 37 °C for one hour. PBS solution was used as a negative control for 0% hemolysis, and 2% Triton X-100 was used as a positive control for 100% hemolysis. Finally, the well plates were centrifuged at 3500 rpm at 4 °C for 10 min. The supernatant was aspirated, and the absorbance was measured at 490 nm using an enzyme marker. Each concentration was repeated six times. The hemolysis rate was calculated according to the following formula:

Hemolysis rate (%) = (OD_test_ − OD_PBS_)/(OD_Triton X-100_ − OD_PBS_) × 100% (2)

### 2.8. Cell Cytotoxicity of the Hydrogel

The cytotoxicity of the hydrogel was tested on mouse fibroblasts (L929 cell line), using CCK-8 and Live/Dead assays. L929 cells were first seeded into 96-well cell culture dishes (1 × 10^4^ cells/well), and the cells were incubated overnight at 37 °C in a CO_2_ incubator. The RD-Gel/Gel extracts were prepared by adding the hydrogel into the RPMI-1640 medium in a constant-temperature oscillating incubator at 37 °C for 48 h, obtaining the supernatant after centrifugation. The extracts were diluted to different concentrations with fresh medium when used. The original medium was removed, and the cells were then treated with different concentrations of extraction solution of hydrogels for 48 h; the control group was treated with the same volume of RPMI-1640 medium. Following incubation, the medium was discarded, and the cells were treated with CCK-8 (10% v/v in RPMI 1640 medium) solution for one hour in the dark. The absorbance at 450 nm was measured using an ELISA Reader. Six replicate wells were set up for each group. The cell viability was calculated based on the absorbance values of the experimental wells.

Cells incubated with the drug were stained using a Live/Dead® Viability/Cytotoxicity Assay Kit (Beyotime, Shanghai, China). Extraction solutions of hydrogels were incubated with cells in 24-well cell culture plates for 48 hours followed immediately by incubation in PBS containing Calcein AM and PI (propidium iodide) for 30 min. Samples were visualized by confocal laser scanning microscopy and live cells were stained with Calcein-AM (green) and dead cells with PI (red).

### 2.9. Antioxidative Effect of the Hydrogel

The ability of hydrogel to scavenge free radicals (1,1-diphenyl-2-picrylhydrazyl, DPPH) was measured. A solution of 0.0178 mmol/L DPPH was prepared and stored in the dark. Gel and RD-Gel hydrogels were extracted in 2 mL of ethanol solution for 48 h. Subsequently, 200 μL of hydrogel extraction was added into 2.8 mL of DPPH ethanol solution, followed by incubation in the dark for 30 min, and the sample was centrifuged and aspirated. The absorbance value of the supernatant was measured at 516 nm. The scavenging rate of DPPH· by hydrogel was calculated according to the following formula:

DPPH clearance rate (%) = (A_0_ − A_t_)/A_0_ × 100% (3)

where A_0_ is the absorbance value of DPPH ethanol solution without the addition of hydrogel, and A_t_ is the absorbance value of the DPPH ethanol solution incubated with the hydrogel.

The intracellular ROS scavenging ability of the hydrogel was tested on the L929 cell line using the Reactive Oxygen Species Assay Kit (Beyotime, Shanghai, China). Cells were inoculated in 24-well plates and incubated for 24 h, followed by a further 30 min incubation with different groups of extracts. After rinsing with PBS, cells were incubated with DCFH-DA (10 μM, 1640 without FBS) for 20 min. Intracellular ROS levels were assessed by confocal fluorescence microscopy photography.

### 2.10. Determination of Anti-Inflammatory Effect of the Hydrogel

RAW264.7 macrophages were inoculated in high-sugar DMEM (10% FBS), then placed in an incubator at 37 °C with CO_2_. After the cells fused to 60~80%, an LPS (1 μg/mL) solution and hydrogel extract were mixed and added to the RAW264.7 cells for 24 h incubation. The supernatant was collected, followed immediately by centrifugation at 1000 rpm for 5 min and storage at 4 °C for assay. ELISAs of IL-6, IL-1α and TNF-α were performed according to the kit’s instructions. The amounts of the cytokines were calculated based on the readings of the samples and the calibration curves.

### 2.11. Hemostatic Ability of the Hydrogel In Vivo

A rat liver hemorrhage model was established to assess the hemostatic ability of the hydrogel. Firstly, the rats were anesthetized, a transverse incision was made in their abdomen to expose the liver, and the surrounding blood was carefully removed using filter paper. The liver was placed on pre-weighed filter paper (W_0_), and an 18G needle was used to pierce the liver and cut a cross to make it bleed. Commercial hemostatic sponges Merocel, Gel, and RD-Gel were placed on the bleeding site immediately after the cut, and untreated wounds were set as controls. Each group contained 3 rats. The bleeding sites were photographed at 0, 30 and 60 s. The weight of the filter paper (W_t_) at 60 s after cutting was weighed. Blood loss was calculated as the difference in mass of W_t_ − W_0_.

### 2.12. In Vitro Cell Migration Assays of the Hydrogel

The effect of hydrogel on the migration of L929 fibroblast cells was investigated using a scratch wound healing assay [23]. L929 was inoculated in 12-well cell culture dishes (30 × 10^4^ cells/well) and incubated in a CO_2_ incubator at 37 °C for 24 h. Then, the cells were scratched with sterilized 20 μL pipette tips to form incisions and washed 3–4 times with PBS to remove cell debris. Immediately afterwards, cells were co-incubated with RD-Gel/Gel hydrogel extract in RPMI-1640 medium supplemented with 2% FBS. The RD-Gel/Gel extracts were prepared by adding the hydrogel into the RPMI-1640 medium in a constant-temperature oscillating incubator at 37 °C for 48 h, obtaining the supernatant after centrifugation. The extracts were diluted to different concentrations with fresh medium when used. The scratch area was photographed and processed with ImageJ software to calculate the wound area. The reduced wound area (%) was calculated according to the following formula:

Reduced wound area (%) = A_t_/A_0_ × 100%. (4)

where A_0_ and A_t_ represent the initial area and the area of the cell being cultured for 12 and 24 h, respectively.

### 2.13. Wound Healing Efficacy of the Hydrogel In Vivo

To establish the model of complex burn wound on animals, SD rats (male, three weeks old) were randomly divided into three groups (n = 5). Each rat was weighed, anesthetized, dorsally shaved, and burned using the acid–base scalding method. A 3-layer gauze block (2 cm in diameter) soaked in 2.5 mol/L NaOH was placed on both sides of the rat’s back and scalded with certain pressure for 60 s, resulting in deep second-degree burns. Immediately afterwards, a full skin lesion of 1.5 cm in diameter was cut with a Skin Punch. PBS, RD-Gel, and Gel dressings were applied on the animals in each group. Photographs of the wound area were taken on days 3, 7, and 14 and analyzed with ImageJ software. The degree of wound closure was calculated using the following formula:

Reduced wound area (%) = A_t_/A_0_ × 100%. (5)

where A_0_ and A_t_ are the wound area at day 0 and the specified time point, respectively. On days 3 and 14, wound tissue was collected for further analysis.

### 2.14. Histopathological Analysis

The sheared wound tissue was fixed overnight in 4% paraformaldehyde. It was embedded in paraffin wax and then cut to a thickness of 5 μm. The method using hematoxylin–eosin and Masson’s trichrome staining was adopted to observe histological changes of the collected tissues.

### 2.15. Statistical Analysis

All data are expressed as the mean ± SD (n ≥ 3). Data between or within groups were assessed for significant differences using analysis of variance for multiple comparisons (Prism8.0 GraphPad, USA). Significant differences between two groups are indicated with an asterisk (^∗^ *p* < 0.05, ^∗∗^ *p* < 0.01, ^∗∗∗^ *p* < 0.001).

## 3. Results and Discussions

### 3.1. Micronizing the Resin and Determination of the Content of Loureirin A and Loureirin B in Resina Draconis

**Figure 1:**
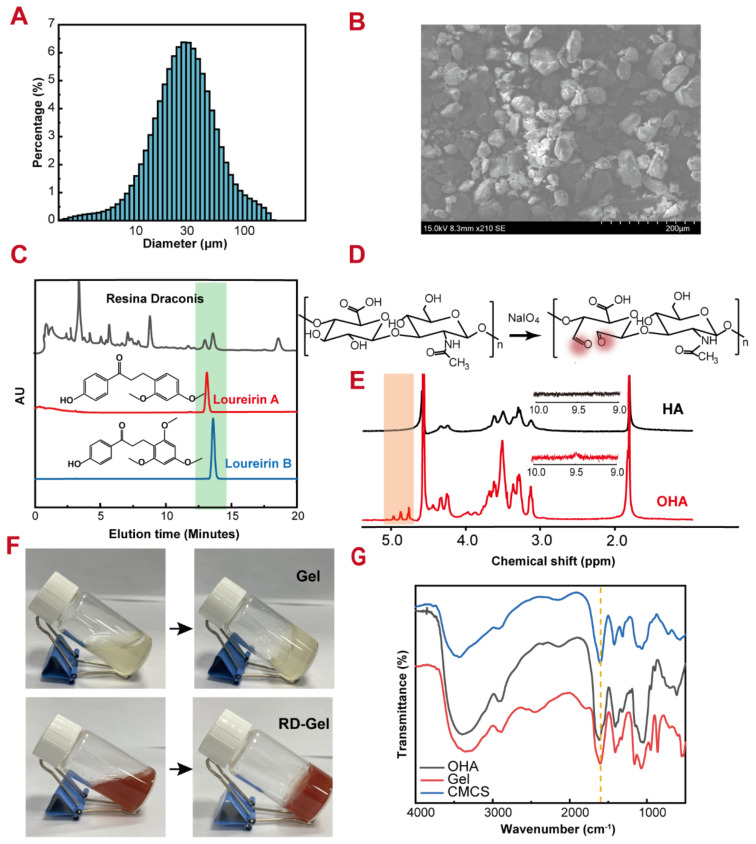
Characterizations of Resina Draconis and synthesized hydrogels. (**A**) Particle size distribution of Resina Draconis. (**B**) SEM image of Resina Draconis. (**C**) Liquid chromatogram of Resina Draconis, Loureirin A and B. (**D**) Synthesis route of OHA. (**E**) ^1^H-NMR spectra of HA. (**F**) Formation of Gel and RD-Gel. (**G**) FT-IR spectra of OHA, CMCS, and the formed Gel.

A micronizer was used to crush the resin into fine powders. After a few rounds of crushing, the size of the resultant particles was measured to be uniformly distributed at around 31 μm (Figure 1A). An SEM graph further showed the morphological appearance of the particles (Figure 1B). Loureirin A and Loureirin B are the two major active compounds in Resina Draconis. The HPLC method was used to determine the contents of Loureirin A and Loureirin B (Figure 1C). The standard curve of Loureirin A and Loureirin B showed satisfactory linearity in the range of 1.5 to 60 μg/mL (Figures S1 and S2). After dissolving the resin in methanol and measuring the compounds using the HPLC method mentioned above, the content of the Loureirin A and Loureirin B in the resin was calculated to be 26.04 mg/g and 149.93 mg/g, respectively. The release of Loureirin A and Loureirin B from the particles was also investigated. Both of Loureirin A and Loureirin B reached approximately 80% release after 120 h of incubation in the release medium (Figure S3).

### 3.2. Formation of RD-Loaded Hydrogels

HA, a constituent of the extracellular matrix with good compatibility with the skin, and CMCS with good biodegradability were chosen as the main materials to prepare the hydrogel. Firstly, OHA was obtained by a ring-opening reaction through the oxidation of HA with NaIO_4_, which allowed HA to acquire two aldehyde groups (Figure 1D). Compared with the ^1^H-NMR spectra of HA, two newly formed peaks at 4.8 ppm, 4.9 ppm and 5.0 ppm were observed on that of OHA, corresponding to the hemiacetal protons and vicinal hydroxyl groups. The peak at 9.5 ppm indicates the aldehyde group of OHA after the oxidation of HA by sodium periodate (Figure 1E). Meanwhile, the infrared data showed that the new absorption peak at 1724 cm^−1^ was the stretching vibration absorption peak of -C=O in the OHA aldehyde group formed after the partial epoxidation of HA (Figure S4). The oxidation degree of OHA was measured as 32% by a hydroxylamine hydrochloride method. The gel was obtained by mixing 4% of OHA and 4.5% of CMCS solutions by vortex in the ratio of OHA:CMCS = 1:3, whereas RD-Gel was obtained by uniformly dispersing Resina Draconis particles into the OHA solution in advance and then mixing with the CMCS solution (Figure 1F). The time to form gel was determined by a tube-inversion method; notably, the gel formation time of the drug-laden hydrogel was significantly shortened, to 40 s, compared with 53 s for the unloaded hydrogel, which may be due to the addition of the Resina Draconis powder decreasing the flowability and aiding the formation of the gel. Rapid gel formation is beneficial for burn wounds because it covers the wound area quickly and prevents further wound bleeding and contamination.

The hydrogels were formed by OHA and CMCS via the Schiff base reaction between the aldehyde groups in OHA and the amino groups in CMCS. FT-IR spectra of the hydrogels were measured to confirm their formation. The results showed the typical peaks of the groups on OHA and CMCS molecules (Figure 1G). For instance, the broad peak at 1643 cm^−1^ in the gel contained the asymmetric stretching vibration peak of the -COO group peak in CMCS (1606 cm^−1^) and the C=O and N-H stretching vibration peaks of the amide I band in OHA (1626 cm^−1^).

### 3.3. Characterizations of Hydrogels

**Figure 2:**
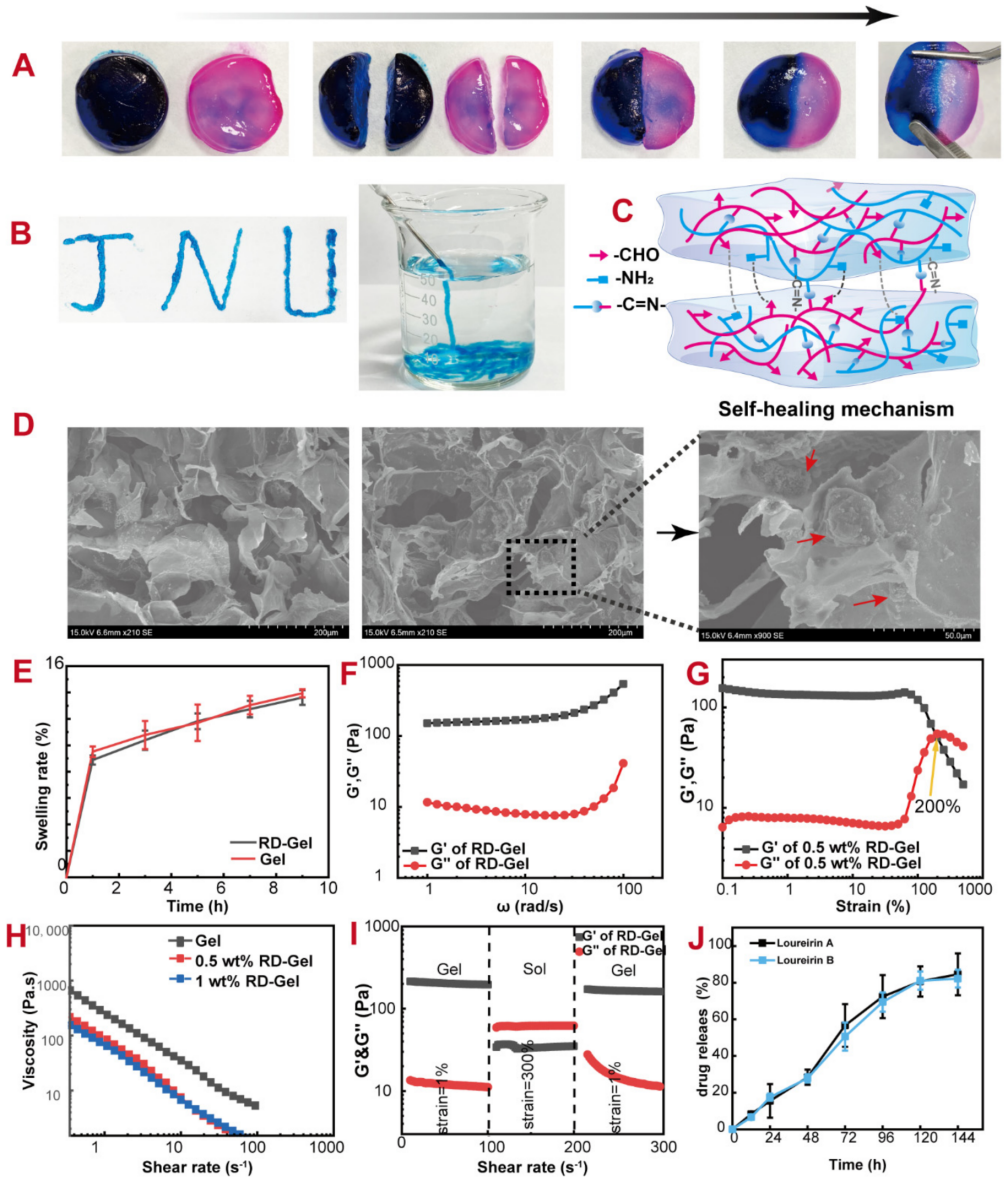
Characterizations of the prepared hydrogels. (**A**) Demonstration of the self-healing property of hydrogels. (**B**) Injectability of the hydrogel. (**C**) Schematic illustration of the reversible dynamic reaction of Schiff base bonds in hydrogel. (**D**) Morphological structure of the hydrogels visualized by SEM, scale bar: 200 μm and 100 μm. The red arrows represent Resina Draconis. (**E**) Swelling rate of hydrogels. (**F**) Frequency sweep of RD-Gel. (**G**) G′ and G″ of the hydrogel on strain sweep. (**H**) Shear viscosity tests of the hydrogels. (**I**) Rheological properties of the hydrogels in the test of changing strains. (**J**) Release of Loureirin A and Loureirin B from the RD-Gel in vitro.

One of the advantages of the prepared RD-Gel was its self-healing property, which allowed the gel to remain relatively integrated even when being stretched or temporarily broken. Figure 2A shows that two-piece hydrogels with different colors were cut and rejoined together for approximately 2 to 3 min, and the hydrogel bonded together and could be stretched without showing any signs of breakage. This originates from the nature of imine bonding of Schiff base, which is in the dynamic balance of breakage and formation, showing macroscopic regeneration under mild reaction conditions, for instance in a physiological pH environment and under room temperature (Figure 2C). Furthermore, Figure 2B shows that the gel could easily be injected through a needle from a syringe, which implied that the gel could be conveniently applied on either various surfaces or cavities for treatment.

The morphology of the lyophilized hydrogels was observed using SEM. The porous and loose morphology of the gel was not affected after drug loading, and uniformly dispersed Resina Draconis particles were observed in the three-dimensional network of the gel (Figure 2D). Burn wounds are often accompanied by blisters and exudate; therefore, hydrogels with good water absorption properties are essential. This porous structure facilitates the rapid absorption of large amounts of exudate from the wound surface and allows the delivery of drug molecules and the transport of oxygen [24]. The water absorption of the hydrogels was studied by incubating them in PBS at 37 °C for 9 h (Figure 2E). It can be observed that both the blank and drug-loaded gels rapidly absorbed buffer solution in the first hour and gradually absorbed more water and gained roughly 15% weight of the original gel in the following 8 h. This property of the prepared gel to maintaining a moist environment close to the physiological state is very important for wound recovery, which promotes the growth of granulation and facilitates the division of skin cells, ultimately leading to complete wound healing.

### 3.4. Rheological Characterizations

After applying the hydrogel on the therapeutic locations, interactions within the hydrogel are one of the key factors affecting cell growth or drug release. Changes in the internal structure of the hydrogel are closely related to rheological properties such as material viscosity and elasticity. The rheological parameters generally tested for hydrogels are G′ and G″. The elastic modulus characterizes the solid-like behavior of the material, whereas the viscous modulus is the heat dissipation of the material due to deformation when the deformation stops after the removal of the applied force, characterizing the liquid-like behavior of material [25], which is also called the loss modulus.

Figure 2F shows the variation in G′ and G″ with a frequency sweep from 0.1 to 100 rad/s. The higher value of G′ than that of G″, which is about 10 times greater than G″, indicated that the elastic component of the system was dominant and the whole system was a typical viscoelastic hydrogel [26]. Figure 2G shows the trends in G′ and G″ intersected at 200% strain. When the strain pressure continued to increase, a sudden decrease in G′ and an abrupt increase in G″ was observed, which indicated the collapse of the internal structure of the hydrogel, transitioning from the state of solid with gel properties to the state of liquid with fluid properties. Moreover, lower RD-loaded gel of 0.5 wt.% showed a higher critical strain than the 1.0 wt.% RD-Gel (Figure S5), indicating that the 0.5 wt.% RD-Gel can withstand greater deformation. This may be due to the fact that a higher content of particles occupied more internal space of the hydrogel and hampered the formation of Schiff base cross-linking between the amino group of OHA and the aldehyde group of CMCS, finally reduced the cross-link density of the hydrogel.

The results of the viscosity property of the hydrogels were shown in Figure 2H. The hydrogels exhibited shear thinning behavior over a range of shear rates. Loaded RD particles also influenced the viscosities of the hydrogels, especially for the RD-Gel sample with a higher RD content of 1.0 wt.%. Higher viscosity would be beneficial for the RD-Gel when it is applied on the wound, because this could aid the dressing to adhere to the tissue and prevent trauma. Based on these considerations, RD-Gel of 0.5 wt.% drug loading with better mechanical strength and higher viscosity was chosen for the subsequent studies.

When normal hydrogel-based dressings were applied on the wounds, most of them tended to be deformed or even broken upon external force, due to their rigid and non-dynamic nature of the constituent skeletons, which compromised their therapeutic effects. Prepared RD-Gels overcame this limitation of normal gel and demonstrated self-healing properties. Figure 2I shows the changes in G′ and G″ for RD-Gel at strains from 1% to 300%. The continuous shear strain varied alternately between 1% and 300%. At a low shear strain of 1%, the G′ was significantly higher than the G″, and the stress in each segment remained stable for 100 s. At a high shear strain of 300%, the hydrogel showed a shear thinning property. G″ was higher than G′, and G′ and G″ changed synchronously with strain. This transition of the elasticity and fluidity was repeatable, which confirmed the self-healing of the hydrogel.

### 3.5. In Vitro Drug Release of the RD-Gel

Ideal drug-loaded dressings would be able to release therapeutic entities during the periods of applications. Furthermore, proper drug release from the dressings would be very favorable for the treatment of burn wounds. To investigate the in vitro release of drugs, hydrogel samples were immersed in the release media in an oscillating incubator at 37 °C and marker compounds, Loureirin A and Loureirin B were measured to represent the released active components of the RD-Gel. Figure 2J shows the results of the in vitro release experiment. Generally, the Loureirin A and Loureirin B were released steadily, almost following a kinetics of zero-order. The release of Loureirin A and Loureirin B reached roughly 55% at 72 h; then, the release rate gradually slowed down and reached approximately 80% at 120 h. Notably, Loureirin A and Loureirin B showed very similar release profiles because they have quite similar chemical structures and lipophilicities. In general, the release profiles of the RD-Gel were ideal; the drugs were released steadily and lasted for 4–5 days. This sustained release formulation improved the release of the active compounds from the RD and provided a more durable solution of dressing for the burn wounds, which may favor the key period of wound recovery processes (hemostasis, inflammation, proliferation, and remodeling) [27].

### 3.6. In Vitro Biocompatibility Tests of RD-Gel

**Figure 3:**
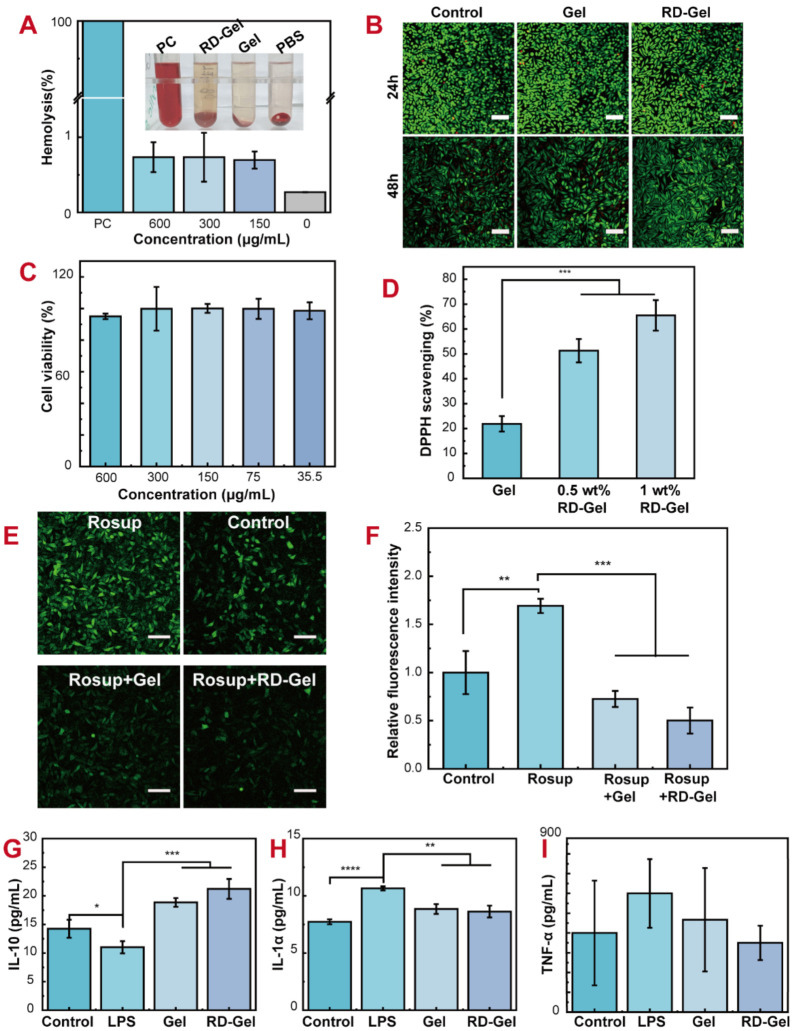
Cytocompatibility, antioxidant and anti-inflammatory properties of hydrogels in vitro. (**A**) Hemolytic test of different hydrogels with blood samples. The X-axis refers to the concentrations of the RD-Gel co-incubated with the blood cells. (**B**) Cytocompatibility of hydrogels indicated by live/dead staining. Scale bar: 100 μm. (**C**) Cell viability of L929 following incubating with hydrogel extracts for 48 h. (**D**) DPPH scavenging percentage for the treatments with Gel and RD-Gel. (**E**) The intracellular ROS level indicated by DCFH-DA probe. Scale bar: 100 μm. (**F**) The quantitative analysis of intracellular ROS depletion. (**G**) Expression of inflammatory factors: IL-10. (**H**) Expression of inflammatory factors: IL-1α. (**I**) Expression of inflammatory factors: TNF-α. For statistical analysis, ∗ represents *p* < 0.05, ** represents *p* < 0.01, *** represents *p* < 0.001, **** represents *p* < 0.001.

The biocompatibility of biomaterials is a major concern when used in clinical dermatology, as they may cause irritant skin, cell damage, or even hematotoxicity. Hemolysis, leukocyte activation, and platelet adhesion and activation, especially for burns, often happen where frequent dressing changes initially bring the dressing into repeated contact with the wound blood [28]. Therefore, hemocompatibility is considered one criterion for the clinical suitability of biomaterials that allow blood contact. Figure 3A shows the result of the biocompatibility test of the prepared RD-Gel co-incubated with rat blood. Clear supernatants were observed in all groups compared with the positive control group with Triton X-100, which showed a completely red color. The hemolysis rate was measured and calculated to be less than 1% in all experimental groups, indicating that the RD-Gel has good hemocompatibility and is considered safe when it directly contacts with burn wounds.

The cell viability and proliferation were analyzed by live/dead staining. As shown in Figure 3B, after 24 and 48 h incubation, most of the cells in the experimental and control groups expressed green fluorescence, and only a few dead cells appeared after 48 h of incubation (red fluorescence). The cytotoxicity of the RD-Gel was further investigated using a CCK-8 assay. The hydrogel showed almost no cytotoxicity to L929 fibroblasts after 48 h incubation with different concentrations of hydrogel extracts (Figure 3C). The above results indicated that RD-Gel hydrogels have good biocompatibilities when contacted with blood or cells related to open wounds, which may be due to the fact that both HA and CMCS are natural biological macromolecules. HA is a key component of the extracellular matrix (ECM), usually found in skin tissue, mammalian bone marrow, articular cartilage and synovial fluid, and plays roles to interact with cells and the ECM [29]. Chitosan is also from natural sources and widely used in biomedical materials due to its degradability and low toxicity [30,31]. The combination of these two biomaterials guaranteed the safety of the in vivo studies of the prepared RD-Gel.

### 3.7. In Vitro Antioxidant Ability and Anti-Inflammatory Efficiency of RD-Gel

After burns, ROS generate considerable amounts of harmful substances and are associated with systemic inflammatory response syndrome, immunosuppression, and tissue damage [32]. Therefore, drugs with free radical scavenging ability are beneficial to be used on wounds. DPPH was used to generate free radicals and to explore the antioxidant effect of RD-Gel (Figure 3D). The Gel group showed a limited antioxidant effect of 20.9%. The RD-Gel group presented higher antioxidant effect (51.3% and 60.7%) and the DPPH scavenging rate was higher in the group with higher contents of RD. The excellent antioxidant effect of RD-Gel comes from a variety of active components in the Resina Draconis, among which loureirin A and loureirin B are flavonoids with multiple hydroxyl groups that can consume free radicals.

Assessment of intracellular levels of oxidative stress after hydrogel treatment was performed using a DCFH-DA probe method. As shown in Figure 3E,F, intracellularly produced ROS were labeled by the probe as green fluorescence. The fluorescence was significantly enhanced and increased 1.7-fold after the cells were treated with Rosup, an ROS stimulating reagent. When the stimulated cells were incubated with the RD-Gel, a clear decrease of 70% fluorescence intensity was observed compared with the non-treatment group. Blank Gel also showed considerable effect to decrease the ROS production, showing that both the blank Gel and RD-Gel effectively alleviated the intracellular oxidative stress.

The healing process is regulated by a variety of growth factors and cytokines that are released from the wound site [33]. Dysregulation of the immune response usually contributes to impaired healing [34]. It is therefore particularly important to inhibit the excessive release of pro-inflammatory factors and to upregulate anti-inflammatory factors. Pro-inflammatory mediators, such as tumor necrosis factor alpha (TNF-α) and interleukin 6 (IL-6), can be upregulated during the inflammatory response [35]. Figure 3G–I shows the in vitro anti-inflammatory effects of RD-Gel incubated with LPS-stimulated RAW264.7 cells [36]. After treatment with RD-Gel, the levels of TNF-α and IL-1α, as the biomarkers of pro-inflammatory cytokines, decreased significantly. On the other hand, the level of IL-10, as the biomarker of anti-inflammatory cytokines, increased by roughly 110%. The results confirmed that RD-Gel can downregulate typical pro-inflammatory cytokines and upregulate certain anti-inflammatory cytokines. Excessive inflammatory reactions in burn wounds can exaggerate damage of the wound tissues and delay the healing processes. The anti-inflammatory effect of RD-Gel would promote rapid passage through the inflammatory phase of the wound and avoid excessive inflammatory reactions causing local tissue damage.

### 3.8. Hemostatic Effect of RD-Gel In Vivo

**Figure 4:**
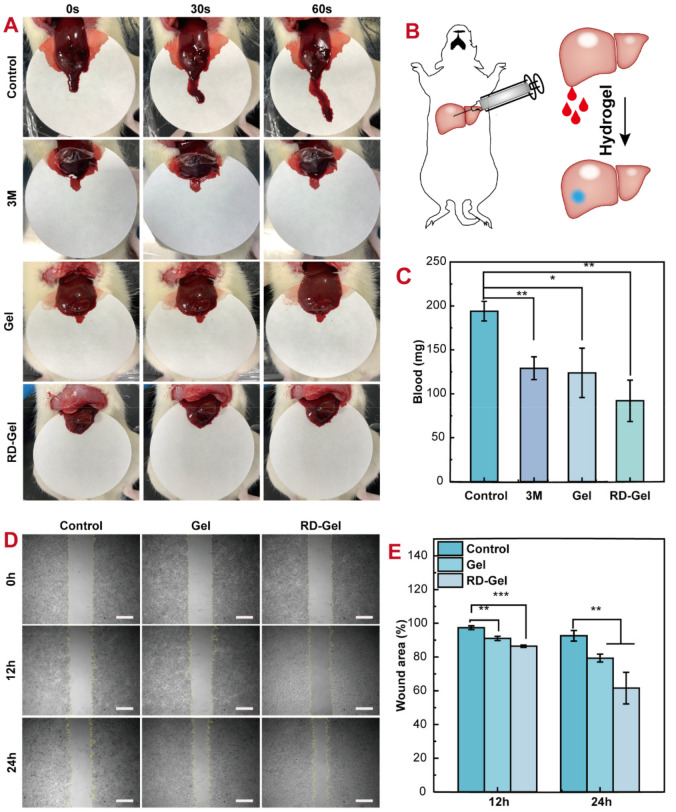
(**A**) Representative photographs of bleeding livers following different treatments at predetermined time points. (**B**) A schematic illustration of a liver hemorrhage model. (**C**) Total mass of blood loss in 60 s from punctured livers. (**D**) Representative photographs of scratch wounds at 0 h, 12 h, and 24 h following the treatments with control, Gel, and RD-Gel. Scale bar: 100 μm. (**E**) Wound area calculated (%) at 0 h, 12 h, and 24 h. For statistical analysis, * represents *p* < 0.05, ** represents *p* < 0.01, *** represents *p* < 0.001.

Complex burn wounds are normally mixed damages with various depths and severities, most of which accompany damaged vessels and bleedings. The ability of instantaneous hemostasis is an important indicator to evaluate wound dressings, which are often needed to cover bleeding areas to prevent excessive blood loss. A liver bleeding model was used to test the hemostatic ability of RD-Gel, with lower bleeding indicating superior hemostatic ability. When the precursor solution was applied to the wound, the solution immediately mixed with the blood and rapidly became gel and adhered to the wound surface. The bleeding stopped immediately and no sustained bleeding from the wound was observed for the next 30 and 60 s for gel samples (Figure 4A). Figure 4B illustrates the experimental setting for the hemostasis experiment. The results in Figure 4C showed that the highest bleeding was observed in the control group with 194 mg, while all dressings significantly reduced the bleeding. Especially, the weight of the bleeding was weighted to be roughly 92 mg for RD-Gel group, only 47% compared with the control group. The excellent hemostatic performance may be due to the three-dimensional porous structure of the hydrogel, which rapidly absorbed large amounts of blood in the wound while physically blocking the bleeding point at the wound to stop further blood loss due to its strong tissue adhesion, followed by the immediate aggregation of platelets at the site of injury to initiate local and coagulation cascade activation, leading to fibrin clot formation and rapid advancement of the wound to the next stage [37].

### 3.9. Migration-Promoting Effects of RD-Gel

The restoration of barrier function in wounded skin requires successful migration, proliferation and differentiation of keratinocytes, a process known as re-epithelialization [38]. An ideal dressing would be able to promote the migration of the cells to accelerate the closing of the open wounds and regrowth of the fresh tissues. The role of hydrogel in promoting wound healing was explored and the results are shown in Figure 4D,E. Compared with the control group, Gel and RD-Gel groups promoted cell migration to varying degrees. At 24 h, wound closure rates were reduced by 13% and 31% for Gel and RD-Gel groups, respectively, indicating that the hydrogel and Resina Draconis significantly promoted cell migration. Cell migration is an important part of the proliferative phase of wound healing and is accompanied by the proliferation and differentiation of dermal fibroblasts [39]. The acceleration of this step helps to shorten the lengthy wound remodeling phase, which usually lasts for several weeks or months [40].

### 3.10. Wound Healing Performance of Hydrogel In Vivo and Histological Analysis

**Figure 5:**
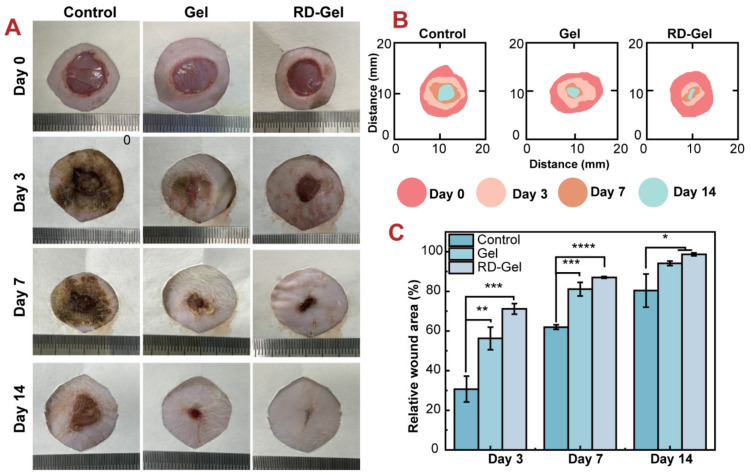
(**A**) Photographs of wounds on model animals with PBS, Gel, and RD-Gel at day 0, 3, 7, and 14. (**B**) simulation of the wound area on different days. (**C**) Statistical results of the remained wound area (%) of each group on different days. For statistical analysis, * represents *p* < 0.05, ** represents *p* < 0.01, *** represents *p* < 0.001, **** represents *p* < 0.001.

The efficacy of hydrogel on wound healing on rats was investigated, and the results are shown in Figure 5A. The model of complex burn wounds was established by combining a greater mild burn area centered with a deep severe wound area to better mimic real burn wounds with different parts of severities. A greater area of scald was created by applying alkaline solutions on the skin, followed by the creation of a full skin defect wound in the center, thus creating a complex model of burn wounds. After treating with PBS or hydrogels, the wound healing was observed and recorded on days 0, 3, 7, and 14. During the healing process, fresh tissues grew gradually from the wound edges. On Day 3, compared with the control group, the Gel and RD-Gel groups showed cleaner wound surfaces, possibly due to the hemostatic and anti-inflammatory effects of the hydrogels, which inhibited the bleedings and inflammatory infiltration with local exudate. Moreover, the wound closure rate was recorded and calculated to be 56.3% and 76.2% for Gel and RD-Gel groups on day 3, respectively. On day 7, the differences among the groups were greater, a 19.1% increase in Gel closure and a 25.1% increase in Rd-Gel closure compared with the control group were recorded (Figure 5B,C). After 14 days of treatment, the hydrogel groups showed good healing effects, especially the wounds treated with RD-Gel almost completely healed.

**Figure 6:**
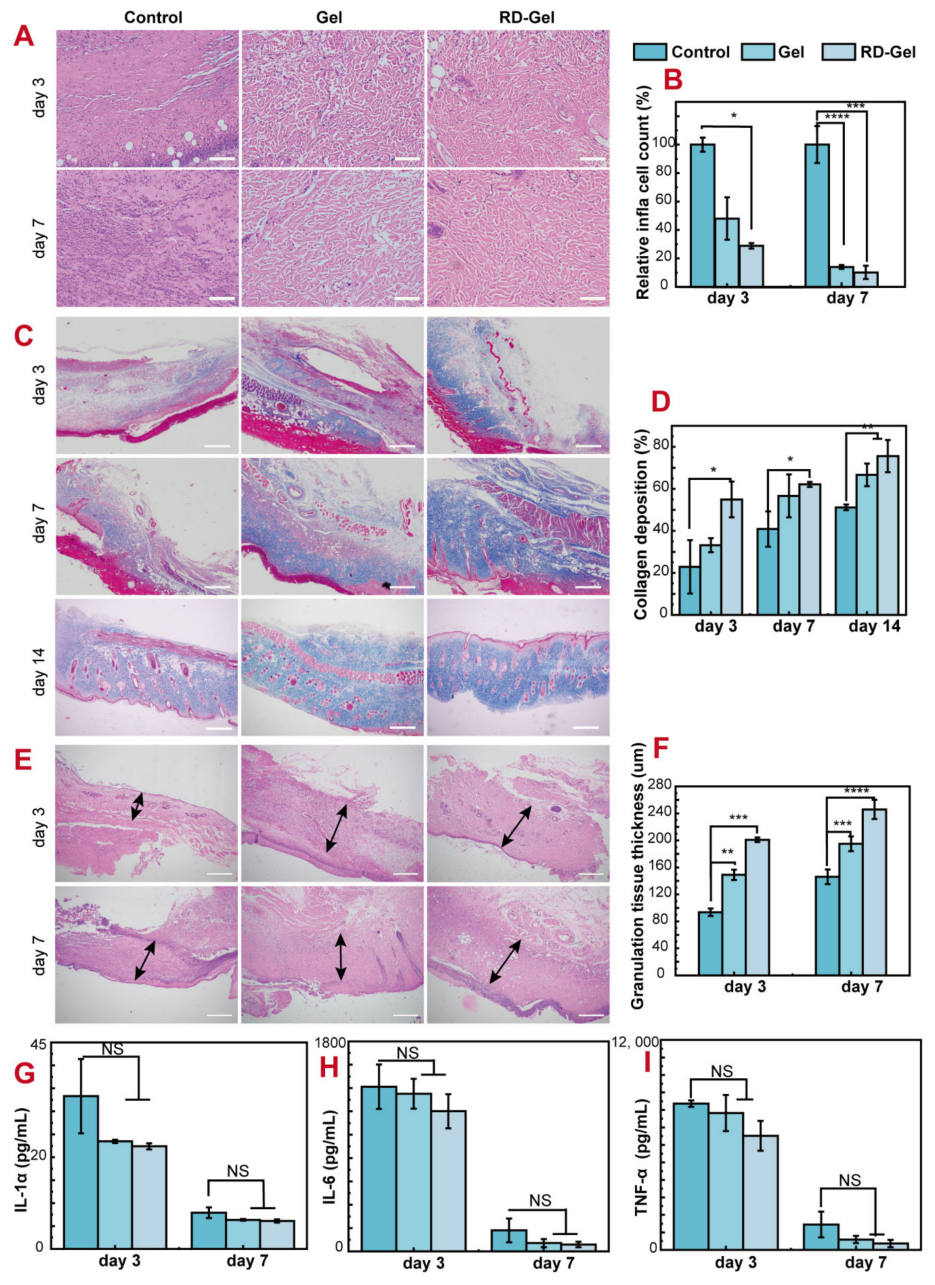
(**A**) H&E-stained images showing inflammatory cell infiltration in the wound area at 3 and 7 days after different treatments. Scale bar: 500 μm. (**B**) Relative number of inflammatory cells. (**C**) Masson’s trichrome staining images of the wound tissues following different treatments for 3 and 7 days. Scale bar: 100 μm. (**D**) Collagen deposition levels in the wound tissues following different treatments for 3 and 7 days. (**E**) H&E staining images indicating the regenerated granulation tissue for 3 and 7 days (black arrow denotes the granulation tissue). Scale bar: 100 μm. (**F**) Quantification of granulation tissue thickness with different treatments for 3 and 7 days. (**G**) Expression of inflammatory factor IL-1α. (**H**) Expression of inflammatory factor IL-6. (**I**) Expression of inflammatory factor TNF-α. For statistical analysis, * represents *p* < 0.05, ** represents *p* < 0.01, *** represents *p* < 0.001, **** represents *p* < 0.001.

Wound tissues from different periods were collected and histologically analyzed using H&E staining. As shown in Figure 6A, there was a significant infiltration of inflammatory cells at the wound site in all groups on the third day of treatment; this may be due to the migration and infiltration of macrophages and neutrophils (cells stained blue), which was particularly obvious in the control group. However, on the 7th and 14th day of treatment, inflammation was significantly reduced in Gel and RD-Gel groups (Figure 6B). In contrast, the inflammation period in the control group was significantly prolonged. This was attributed to the anti-inflammatory properties of Resina Draconis, which effectively relieved inflammation.

Collagen is the most abundant protein in the body and is the main component of the wound matrix. During the healing process of a wound, collagen deposition and the rearrangement of collagen fibers occur [41]. In Figure 6C, the results of Masson’s trichrome staining show a significant increase in collagen expression in the experimental groups (blue section), especially in the Rd-Gel group, with a recorded collagen deposition of 62.15% after seven days of treatment, much higher than that of 40.87% in the control group (Figure 6D). This proved that the fibroblasts in the wound bed of the treated group started to proliferate intensively and the wound gradually healed.

In the process of tissue injury, in order to replace the necrotic parenchyma, the surrounding immature connective tissue can proliferate to form a red granular soft tissue called granulation tissue [42]. The formation of granulation tissue is a key link in wound repair [43]. The thickness of granulation tissue was further counted by H&E staining (Figure 6E). Compared with the control group, the administration group showed thicker granulation tissue on the third and seventh days, and the RD-Gel group was more significant. On the seventh day, the average thickness of the granulation tissue in the RD-Gel group was 246.02 μm, which was 1.68 times greater than that of the control group (Figure 6F). In conclusion, the hydrogel exerts a comprehensive therapeutic effect by slowly releasing active substances such as Loureirin A, thereby achieving optimal granulation tissue formation and collagen deposition.

Supernatants were taken from rat tissues after grinding to determine the expressions of inflammatory factors in the wound bed. IL-6, IL-1α, and TNF-α, all pro-inflammatory factors, often thought to be secreted in elevated levels in chronic inflammation, were tested in the study [44,45]. Figure 6G–I reveals that at the beginning of treatment, all groups showed overexpression of inflammatory factors, followed by significant decreasing trends on the seventh day after treatment. Inflammatory factor secretion was slightly lower in RD-Gel group than that of Gel group, due to the anti-inflammatory nature of RD. The overall trend of tissue inflammation expression was consistent with the in vitro experiments. This may be due to the anti-inflammatory effects of various effective substances in the Resina Draconis.

## 4. Conclusions

In conclusion, the results of the studies confirmed our hypothesis. RD-Gel with good mechanical and self-healing properties was prepared through Schiff-bonding reactions. After physically encapsulating micronized Resina Draconis particles, the RD-Gel demonstrated sustained-release profiles of Loureirin A and Loureirin B, the major active compounds from the Resina Draconis. In in vitro and in vivo experiments, RD-Gel showed hemostasis, scavenged reactive oxygen species, and regulated the expressions of inflammatory factors in the treatment of burn wounds, enabling the wounds to rapidly pass through the hemostatic and inflammatory phases and enter the proliferative and remodeling phases, and eventually accelerated the whole heal process. These results suggest that RD-Gel can be used as a promising dressing to treat complex burn wounds.

## Data Availability

Not applicable.

## Supplementary Materials

The following supporting information can be downloaded at: https://www.mdpi.com/article/10.3390/pharmaceutics14102087/s1. Figure S1: Standard Curve of Loureirin A. Figure S2: Standard Curve of Loureirin B. Figure S3: Release of LoureirinA and Loureirin B from the Resina Draconis particles in vitro. Figure S4: FT-IR spectra of (a) HA, (b) OHA. Figure S5: G′ and G″ of the hydrogel on strain sweep of 1 wt% RD-Gel.
