# Supplementary material for: Resina Draconis Particles Encapsulated in a Hyaluronic-Acid-Based Hydrogel to Treat Complex Burn Wounds
Source: Pharmaceutics. 2022 Sep 29;14(10):2087. doi: 10.3390/pharmaceutics14102087 (PMC9607235; doi:10.3390/pharmaceutics14102087)
Supplement: Supplementary file 1 [file pharmaceutics-14-02087-s001.zip › pharmaceutics-1933987-supplementary.pdf]

Supplementary materials

Resina Draconis Particles Encapsulated in a Hyaluronic Acid Based Hydrogel to Treat Complex Burn Wounds

*Lijun Xu, Ziqiang Zhou, Yuying Chen, Huangjie Lu, Ping Hu**

College of Pharmacy, Jinan University, Guangzhou 511436, Guangdong, P. R. China

Corresponding author: Ping Hu

Mailing address: Jinan University, No.855, East Xingye Avenue, Panyu Districst, Guangzhou 511436, CHINA

Tel.: +86-18581483142

E-mail: inzahu@hotmail.com, pinghu@jnu.edu.cn

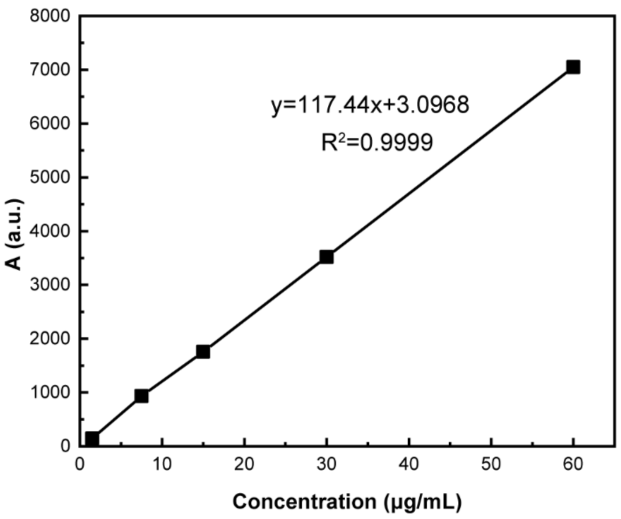

Figure S1: Standard Curve of Loureirin A.

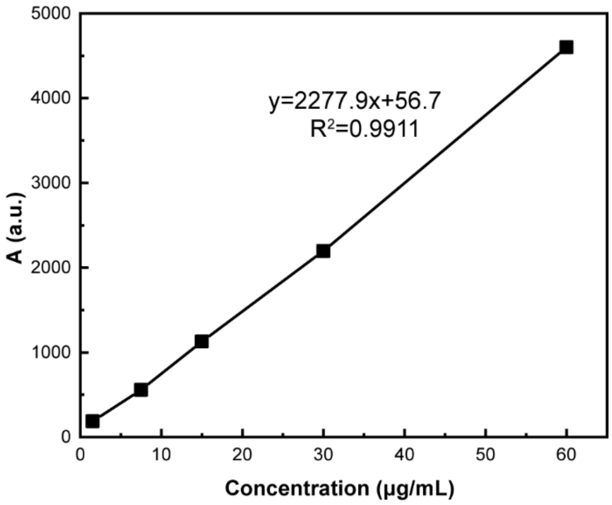

Figure S2: Standard Curve of Loureirin B.

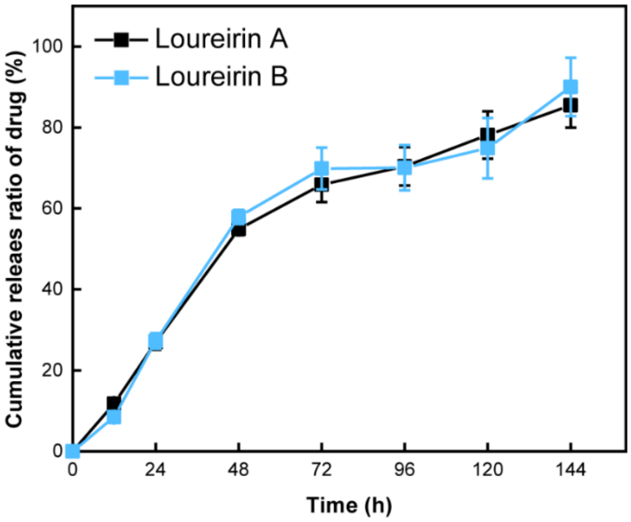

Figure S3: Release of LoureirinA and Loureirin B from the Resina Draconis particles *in vitro*.

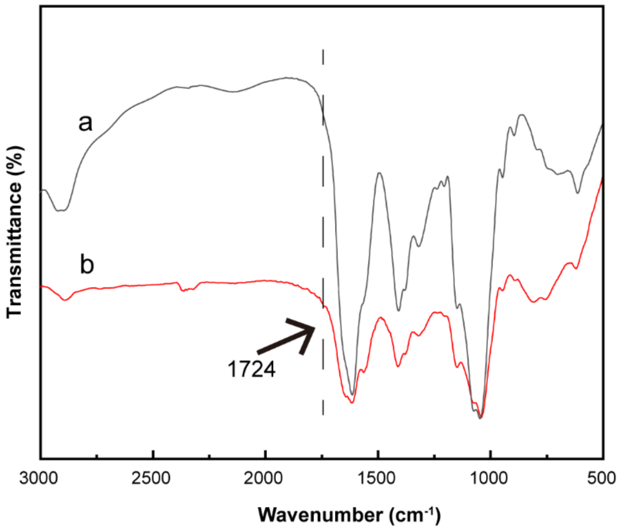

Figure S4: FT-IR spectra of (a) HA, (b) OHA.

Figure S5: ^{1}H-NMR spectra of CMCS.

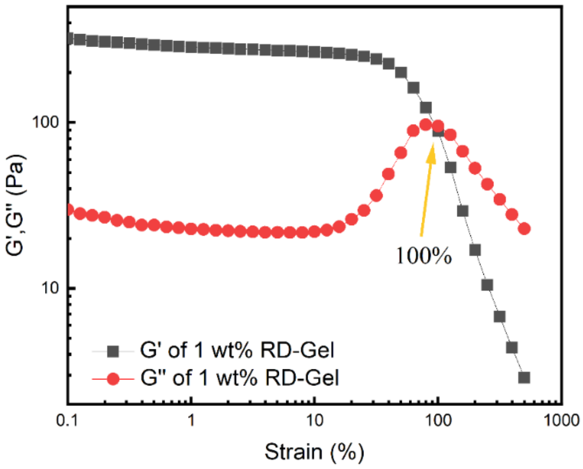

Figure S5: G’ and G” of the hydrogel on strain sweep of 1 wt% RD-Gel.
